# Returning pTau‐217 results: Methods from the Evaluation of Self‐Mediated Alternatives for Risk Testing Education and Return of Results (eSMARTER) study

**DOI:** 10.1002/alz70856_099505

**Published:** 2025-12-24

**Authors:** Claire M Erickson, Kristin Harkins, Elisabeth Wood, Carolyn Langlois, Nicholas J. Ashton, Emily A. Largent, Jason Karlawish, J. Scott Roberts, Angela R. Bradbury, Jessica B. Langbaum

**Affiliations:** ^1^ Banner Alzheimer's Institute, Phoenix, AZ, USA; ^2^ University of Pennsylvania Perelman School of Medicine, Philadelphia, PA, USA; ^3^ University of Pennsylvania, Philadelphia, PA, USA; ^4^ Banner Sun Health Research Institute, Sun City, AZ, USA; ^5^ University of Michigan, Ann Arbor, MI, USA

## Abstract

**Background:**

Though not yet part of routine care, Alzheimer's disease (AD) blood biomarkers are poised for broad use in diagnosis and management and already used in clinical trials to determine participant eligibility. To date, current practices for returning biomarker results focus on implications for trial eligibility, as opposed to risks for AD or treatment side effects. The growing utility of AD blood biomarkers thus requires further development of methods to communicate results. This study builds on prior methods for returning AD risk information (e.g., via *APOE* genotyping, amyloid neuroimaging) by expanding them to plasma pTau‐217 results.

**Methods:**

The Evaluation of Self‐Mediated Alternatives for Risk Testing Education and Return of Results (eSMARTER) is a decentralized, randomized trial to evaluate self‐directed, scalable eHealth platforms for communicating *APOE* and plasma pTau‐217 results. pTau‐217 disclosure will occur approximately six months post‐*APOE* disclosure. Across the US, approximately 600 adults aged 60‐80 will be enrolled (#NCT06459583). The sample will include 400 *APOE4* carriers and 200 non‐carriers. Participants will be randomized to receive results either via telehealth videoconference (led by a healthcare provider) or by eHealth platform (led by the participant). Materials developed for this study include educational materials on AD, pTau‐217, and possible results (negative, intermediate, positive), and disclosure materials for communicating pTau‐217 results and their meaning.

**Results:**

Data collection is ongoing and includes two timepoints (seven days and four weeks post‐disclosure). Results will characterize result comprehension, psychological impact of learning pTau‐217 information, and participant experiences using the eHealth platform.

**Conclusions:**

Disclosing AD biomarker status is becoming more common with the availability of disease‐modifying therapies. Further, the 21^st^ Century Cures Act, which mandates immediate return of most medical tests, coupled with the shortage of genetic counselors and dementia specialists, heightens the need to return results in ways that minimize healthcare system burden. eSMARTER is an opportunity to understand benefits and limitations of remotely returning pTau‐217 results and characterize how people respond to and understand such information. Collection of these novel data will improve return of results processes, expand the types of information returned, and guide future research on returning multiple biomarkers simultaneously.

